# Computational Study of Antibody Binding to SARS-CoV-2 Variants

**DOI:** 10.3390/antib15030043

**Published:** 2026-05-25

**Authors:** Carolyn Chiu, Muhammad Zaki Jawaid, Daniel Lee Cox

**Affiliations:** 1 Department of Physics and Astronomy, University of California, Davis, CA 95616, USA; 2 Epicrispr Biotechnologies, South San Francisco, CA 94080, USA

**Keywords:** SARS-CoV-2, antibody binding, immunity escape, simulation

## Abstract

**Background/Objectives**: The unprecedented structural and binding data for antibodies to the SARS-CoV-2 virus taken together with the mutations for the spike protein allows for a broad simulation study of antibody–spike protein binding. This provides an understanding of the co-evolution of human immunity and viral immunity escape. **Methods**: We utilized the YASARA molecular dynamics program to generate initial structures and simulate to equilibration for six SARS-CoV-2 variants and ten different antibodies sampling two different binding regions to the receptor binding domain of the spike (especially for the Class I antibodies in the same part of the spike that attaches to the ACE2 receptor protein) and one to the N-terminal domain of the spike. Starting structures for antibody binding to variant spike protein domains are perturbatively achieved through point mutations and insertions/deletions in the YASARA program. We employed YASARA to measure interfacial hydrogen bound counts between antibodies and variant spike proteins and the HawkDock MMGBSA program to characterize trends in binding energies with mutation for four of the antibodies. We utilized the VMD program to analyze the time course of hydrogen bond populations. **Results**: As seen in previous studies, interfacial hydrogen bond counts serve as an excellent proxy for binding energies without the large systematic error inherent in the latter. We find that there is generally a decline in antibody binding strength, as measured by interfacial hydrogen bond counts, with viral evolution, but that a modest re-entrance of binding strength is present for most antibodies studied. Generically, the antibody heavy chain binds more strongly to the spike protein, though for approximately half the antibodies the light chain binding strength converges to the heavy chain strength with viral evolution. **Conclusions**: The key conclusion is that the identified re-entrant immunity, speculatively arising from a balancing of maintenance of ACE2-spike binding while escaping antibodies through mutation, allows for some maintenance and even strengthening of immunity for later viral strains from early infection or vaccination.

## 1. Introduction

The SARS-CoV-2 virus engendered a worldwide pandemic from March 2020 to May 2023 that killed tens of millions worldwide. The success of the virus in escaping immune responses through mutations of the SARS-CoV-2 spike protein, which bound both to human angiotensin converting enzyme 2 (ACE2) receptor on the surface of cells and to potentially neutralizing antibodies, contributed to the lethality of disease over time.

Accordingly, there has been considerable experimental and computational interest in understanding which mutations through new strains of SARS-CoV-2 were key to antibody escape. Many of the computational studies were oriented to specific antibody–spike interactions for particular strains of the SARS-CoV-2 virus.

As has been noted elsewhere [1], there is a difficult evolutionary dance the virus plays to escape immunity: most of the effective neutralizing antibodies attach in the same receptor binding domain to which the virus binds to ACE2. Accordingly, a massive immune escape is likely to lead to a virus with weaker cell binding and, presumably, lower cell lethality.

We have adopted a different perspective in this study, which computationally examines the binding strength of ten antibodies covering a broad range of binding sites to the receptor binding domain (RBD) and N-terminal domain (NTD) of the SARS-CoV-2 spike protein and examines six variants of the virus from the original strain of 2020 through the BA.2.86 variant predominant near the end of the pandemic in 2023. The antibodies studied include Class I (binding to the same set of residues in the RBD as the ACE2), Class III (binding to the RBD but away from the ACE2 binding domain), and NTD binding. We are unaware of any study in the literature that has covered such a comprehensive range of antibodies and variants. We omit N-linked glycans from the simulations, which may lead to an overestimate of binding for the Class III and NTD antibodies.

By monitoring the number of interfacial hydrogen bonds between the antibody and the spike protein, we are able to discern some new results relevant to the understanding of the pandemic trajectory.

First, while some antibodies display a monotonic decrease in binding strength with strain evolution, many show a partial re-entrance, that is, the binding strength rebounds at least partially with time so that the antibodies retain some neutralizing capability. We believe this reflects the difficult evolutionary competition between immune escape and maintaining sufficient ACE2 binding. Second, quite uniformly, the heavy chains of the antibody bind more strongly than the light chains. Third, in general, the Class I antibodies bind more strongly than the Class III or N-terminal antibodies studied. Fourth, those Class I antibodies alleged to show higher efficacy for omicron and descendant strains were not found to bind more strongly than the earlier delta or original (wild-type [WT]) strains.

Additionally, as we found in previous studies, the interfacial hydrogen bond count serves as a strong proxy for binding free energy, which we evaluated separately for a representative subset of the antibodies.

The most important emerging qualitative picture from our study is that the viral evolution may provide immune escape from the current extant antibodies, but a global escape from all previous antibodies is likely impossible given that the most efficacious ones bind in the same region as the ACE2, so that high immune escape means weak cellular binding. Also, because immune escape is relative to current extant antibodies, re-entrance in which at there is at least some restoration of immunity from previous antibodies can lead to a persistent robust population immunity to the evolving virus.

## 2. Materials and Methods

### 2.1. Molecular Models

A summary of all the mutations relative to the original (WT) strain in the RBD and N-terminus of the spike protein from the six variants (Delta, BA.1, BA.2, XBB.15, BA.2.86) is found in Table 1.

**Figure 1:**
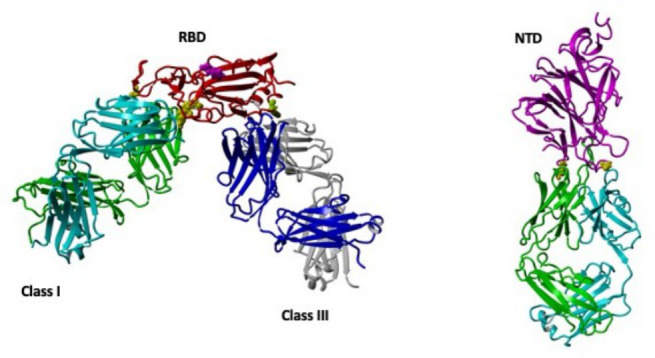
Structures of WT spike protein complexes studied. Binding of RBD (red) to Class I Ab C1A-B12 Fab fragment (binds in ACE2 interface region) [5] and Class III Ab CR.3022 Fab fragment (binds away from ACE2 region) [9]. For C1A-B12, the heavy chain is shown in green, light chain in cyan; for CR.3022, heavy chain is in blue, light chain is in grey. RBD residue away from ACE2 (F347) shown in magenta, residues in ACE2 binding region (R403, S477, Y505) shown in yellow. For CR.3022, we also highlight K386 in chartreuse. Binding of NTD (purple) to Ab 4A8 Fab fragment [10], for which the Ab heavy chain is green, light chain is cyan. For N-terminal binding, residues near interface (K147, D253) shown in yellow. Graphic representations were created with YASARA [11].

We drew starting structures for RBD-ACE2 binding from the Protein Data Bank. Class I antibodies bind in the same region of the RBD as the ACE2. Here, we preferentially use the antibody designations in the literature, but we also refer parenthetically to the Protein Data Bank (PDB) files where the bound structures to relevant RBDs were displayed; P4A1 (7CJF) [4], C1A-B12 (7KFV) [5], 2-15 (7L5B) [6], C1A-C2 (7KFX) [5], C1A-F10 (7KFY) [5], C1A-B3 (7KFV) [5], S2X234 (8ERQ) [7], and Omi-3 (7KF3) [8] were selected to represent the spectrum of Class I antibodies. Class III antibodies bind to the RBD away from where the ACE2 binds and are represented by CR.3022 (6YOR) [9]. For antibodies that bind to the N-terminal domain, we used 4A8 (7C2L) [10], extracting the NTD sequence from one of the full length spike trimers bound to a 4A8 Fab fragment (G14-T299 of the spike protein). Figure 1 shows the structures of representative spike domain–antibody complex types studied in this paper. The chosen antibodies were not comprehensive of all the known neutralizing antibodies for the SARS-CoV-2 spike but summarize a variety of antibodies that target the SARS-CoV-2 virus. We did not study T-cell binding. Antibodies are summarized in Table 2.

N-linked glycans can help shield the RBD and NTD from antibody binding. We have omitted glycans from our simulations. The possible glycan positions for the NTD region are (referencing the WT sequence) N17, N61, N74, N149, N165, N234, and N282. Of these positions, only the N149 is directly at the interface with 4A8, and there was no glycan included in the initial structure determination. For the RBD, there are possible N-linked glycosylation sites at 331 and 343. No glycans were included in the original structure files for the CR.3022 [9] or 2-15 files [6], and a glycan at N343 was included in the starting files for P4A1 [4], C1A-B12,C1A-C2,c1A-F10, and C1A-B3 [5]. The potential impact of omitted glycans is examined in the Discussion.

To adopt starting model structures for studying binding with molecular dynamics, we picked the relevant earliest bound variant spike–antibody structure from the PDB and mutated the residues point by point within the YASARA modeling suite [11]. Three of these structures are shown in Figure 1. When deletions arose, particularly in the N-terminus of the spike, we grafted the corresponding ends within YASARA [11]. Insertions and mutations were built upon starting structures using YASARA’s BuildLoop and SwapRes commands, respectively. While this is basically a perturbative approach biased to the starting structures, unlike an unbiased docking approach, it is unlikely to lead to systematic errors of the kind known in docking protocols. For all but the S2X234 and Omi-3 antibodies, the starting structure is WT. For the S2X234 and Omi-3 antibodies, the starting structures are for the BA.1 strain. Experimental evidence in support of the perturbative approach is discussed in Section 3.

### 2.2. Molecular Dynamics

Simulations of the protein–protein interactions were completed with the molecular-modeling package YASARA [11] by searching for minimum-energy conformations of the SARS-CoV-2-Ab complexes. For each structure, we carried out a energy minimization (EM) routine, which includes steepest descent and simulated annealing minimization to remove clashes and stabilize starting energies to within 50 J/mole.

All molecular-dynamics simulations were run using the AMBER14 force field with Ref. [12] for solute ions, GAFF2 [13], AM1BCC [14] for ligands, and TIP3P for water. The cutoff was 8 Å for Van der Waals forces (AMBER’s default value [15]) and no cutoff was applied for electrostatic forces (using the Particle Mesh Ewald algorithm [16]). The equations of motion were integrated with a multiple timestep of 1.25 fs for bonded interactions and 2.5 fs for non-bonded interactions at T=298 K and P=1 atm (NPT ensemble) via algorithms described in [17]. Prior to counting the hydrogen bonds and calculating the free energy, we carry out several pre-processing steps on the structure, including an optimization of the hydrogen-bonding network [18] to increase the solute stability and a $pK_{a}$ prediction to fine-tune the protonation states of protein residues at the chosen pH of 7.4 [17]. Simulation data was collected every 100 ps after at least 2 ns of equilibrium time, observed via the stabilization of the number of hydrogen bonds, the root mean square deviations (RMSDs), and the interfacial surface area. For all simulations, we require approximately 10 ns or more of equilibrated time as observed by stable values of root mean square deviation (RMSD) from the starting structure.

**Figure 2:**
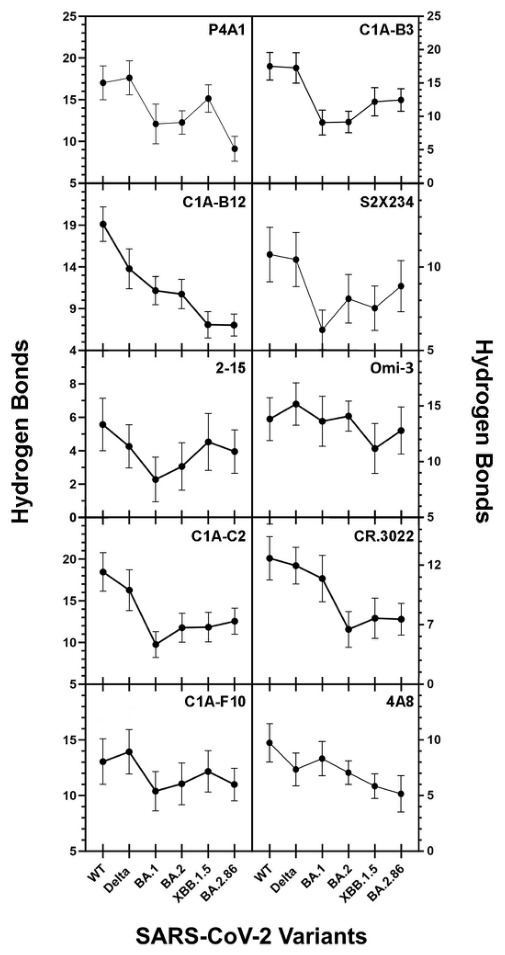
SARS-CoV-2/Ab interfacial hydrogen bond counts. SARS-CoV-2 variants WT, Delta, BA.1 (omicron), BA.2, XBB.1.5, and BA.2.86 plotted for each Ab. BA.2.75 represents BA.2 in 4A8 Ab graph. Note the reemergence effect for most variants where the binding strength rises after falling for subsequent variants. Graphing was performed using GraphPad Prism version 10.0.0 for Windows, GraphPad Software, Boston, MA, USA, www.graphpad.com (accessed on 15 April 2026).

The total interfacial hydrogen bond (H-bond) counts were tabulated using a distance and angle approximation between donor and acceptor atoms as described in [18] and averaged over the equilibration time series of the simulation. Results are shown in Figure 2.

Different equilibrium runs were generated by changing the starting random number seed within YASARA [11].

### 2.3. Endpoint Free Energy Analysis

**Figure 3:**
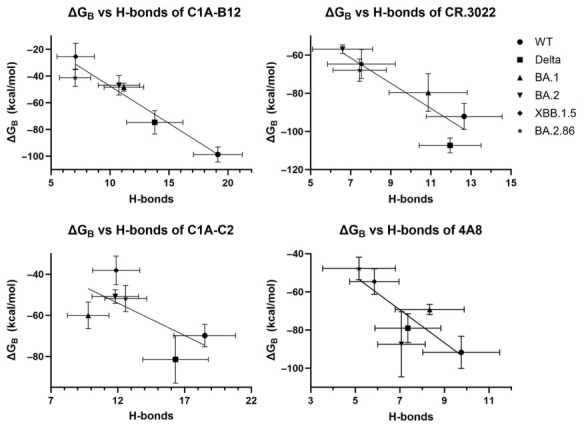
Binding free energy estimate in kcal/mole from GBSA analysis of molecular dynamics equilibrium conformations Class I, Class III, and N-terminal Ab represented. Straight lines were from linear regression, with coefficients of determination for C1A-B12 $R^{2}=0.8713$, C1A-C2 $R^{2}=0.45$, CR.3022 $R^{2}=0.75$, and 4A8 $R^{2}=0.65$. Graphing was performed using GraphPad Prism version 10.0.0 for Windows, GraphPad Software, Boston, MA, USA, www.graphpad.com (accessed on 15 April 2026).

Binding free energy for the energy-minimized structures from molecular dynamics simulations were calculated with the generalized Born surface area (MM/GBSA) method on the HawkDock server [19]. For Class I, Class III, and NTD antibodies, we averaged five snapshots of equilibrium conformations for binding to each SARS-CoV-2 variant. The MM/GBSA approximations overestimate the magnitude of binding free energy in comparison to in vitro experimental estimates, but they correlate strongly with hydrogen bond counts. Correlation plots for endpoint free energy analysis against interfacial hydrogen bond counts are displayed in Figure 3.

### 2.4. Statistical Significance

*T*-tests were performed on every combination of two hydrogen bond means with the same antibody and different spike protein variant. Fifteen combinations were compared for each antibody. For a given combination, their means, standard deviations, and number of points were input into the online *T*-test calculator by GraphPad [20] with the unpaired *T*-test selection. The difference in hydrogen bond counts was statistically significant when the generated *p*-value was less than 0.05. The *p*-value represents the probability that any difference between the two observed groups is due to random chance. A spreadsheet of the *t*-test results are included in the online data.

Because we are running multiple tests (*m* = 15 for each antibody), we can conservatively correct post hoc for significance overestimates in pairwise *t*-tests using the Bonferroni method [21], where we take the *p*-value target value for significance as the single pairwise test of *p* = 0.05 and divide by *m* = 15. Hence, we consider a test significant if the *p*-value falls below $p^{\prime }$ = 0.00333.

### 2.5. Interfacial Hydrogen Bonds Population Analysis

YASARA [11] is effective at counting hydrogen bonds overall, which is a correlate to binding energy. To analyze the population of individual interfacial hydrogen bonds over the course of simulations, we employed a different strategy. First, we transformed the simulation snapshots to a GROMACS [22] file using the mdconvert macro [11]. Second, we uploaded the GROMACS trajectory to the Visual Molecular Dynamics (VMD) viewer [23].

VMD hydrogen bond analyses criteria differ in detail from YASARA. To provide the best match between the two separate programs, we did the following. In the VMD menu, we chose the hydrogen bond analysis macro, with a fixed donor-hydrogen-acceptor (D−H−A) angle cutoff of $\theta_{c}$ = $35^{{}^{\circ}}$ ($\theta_{c}$ is actually $180^{{}^{\circ}}$-〈D−H−A). YASARA softly cuts off for D-H-C anything for $\theta_{c}<80^{{}^{\circ}}$. YASARA imposes a distance criterion dependent upon the H-A distance [18], while VMD measures the D-A distance. Accordingly, we begin with a D-A default distance of 3.5 Å and vary the distance so that the average hydrogen bond count over the equilibrium trajectory matches that determined for YASARA. A full table of resultant hydrogen bond occupancies is available in online data.

## 3. Results

### 3.1. Interfacial Hydrogen Bond Counts

The axis of variant is, effectively, an epidemiological timeline of COVID-19 through the human population.

The most striking aspect of the interfacial hydrogen bond count vs. variant for all but antibodies C1A-B12 and P4A1 is the non-monotonic “re-entrant” behavior of the total hydrogen bond counts. Namely, after some initial decline from the earlier WT and Delta variants, there is subsequently some partial return in interfacial binding efficacy for later variants.

The results of *t*-test show that this re-entrance is statistically significant. We summarize the *t*-test comparisons with the Bonferroni correction for multiple comparisons as follows:

- **P4A1** Differences between BA.1 and BA.2 are not statistically significant ($p^{\prime }>0.0033333$) nor are the differences between XBB.1.5 and BA.2.86. All other differences are statistically significant.
- **C1A-B3** Differences between WT and Delta, between BA.1 and BA2, and between XBB.1.5 and BA.2.86 are not statistically significant, but all other differences are. Hence, the observed re-entrance is statistically significant.
- **C1A-B12** Differences between BA.1 and BA2, and differences between XBB.1.5 and BA.2.86 are not statistically significant, but all others are.
- **S2X234** Differences between WT and Delta are not statistically significant, but all other differences are, so that the observed re-entrance for BA.2.86 is statistically significant.
- **2-15** Differences between Delta and XBB.1.5 and between Delta and BA.2.86 are not statistically significant, but all other differences are, so the observed re-entrance is statistically significant.
- **Omi-3** Differences between WT and BA.1, BA.2, and between BA.1 and BA.2 are not statistically significant, but all others are, so the observed re-entrance observed for XBB.1.5 and BA.2.86 is statistically significant.
- **CA1-C2** Differences between BA.2 and XBB.1.5 are not statistically significant, all others are. Hence the observed re-entrance for BA2, XBB.1.5, and BA.2.86 are statistically significant.
- **CR.3022** Differences between WT and Delta, and between XBB.1.5 and BA.2.86 are not statistically significant, but all others are. Hence the re-entrance for BA.2.86 and XBB.1.5 is statistically significant.
- **C1A-F10** Differences between BA.1 and between BA.2, and BA.2 and BA.2.86 are not statistically significant, but all others are. Hence the re-entrance for BA.2, XBB.1.5, and BA.2.86 is statistically significant.
- **4A8** Differences between Delta and BA.2 are not statistically significant, but all others are. Hence, the re-entrance observed for BA2, XBB.1.5, BA.2.86 is statistically significant.

### 3.2. Binding Free Energy

We performed endpoint free energy analysis for the P4A1, C1A-C2, CR.3022, and 4A8 antibodies. As shown in Figure 3, with the exception of the C1A-C2 antibody, there is a high degree of correlation between the interfacial hydrogen bond counts and the endpoint free energy analysis. Hence, this continues the observation made in Refs. [1,24] that interfacial hydrogen bond counts are good proxies for endpoint free energy analyses. We note that in the case of the C1A-C2 antibody, the poor correlation is driven by the BA.1 variant data point. If that data point is excluded, the $R^{2}$ value improves from 0.45 to 0.70.

The point is important because it can be seen that the binding free energies in Figure 3 are quite large compared to values inferred from typical binding affinity data. As an example, we can take $K_{D}\approx 5$ nM for ACE2-RBD binding from the literature [25]. The dissociation constant $K_{D}$ is given by

(1) $$K_{D}=K_{D0}\exp\left(\Delta G_{B}/\left(RT\right)\right)$$

where standard estimates put $K_{D0}\approx 1$ M (see, e.g., Dill and Bromberg [26]). Solving for $\Delta G_{B}$ gives −11.3 kcal/mole, clearly small in magnitude compared to the values found from GBSA analysis. Assuming the tighter binding of antibodies giving $K_{D}=0.1$ nM changes the estimated $\Delta G_{B}$ to −14.9 kcal/mole, still far below the estimated magnitudes here.

The overestimates of the $\Delta G_{B}$ magnitudes derive from the GBSA approximation itself, where large energies of opposite signs for the entire complex must cancel out to provide the binding energy. For example, for the CR 3022 WT binding, the GBSA contributions are, respectively: Van der Waals: −109.6 kcal/mole, Electrostatic: −302.7 kcal/mole, Generalized Born: 334.7 kcal/mole, and Surface Area: −14.6 kcal/mole. We anticipate the trends of the GBSA binding energy estimates to be accurate, but clearly the absolute values arising from the cancellation of opposing large energies are not.

### 3.3. Population Analysis of Hydrogen Bonds

**Figure 4:**
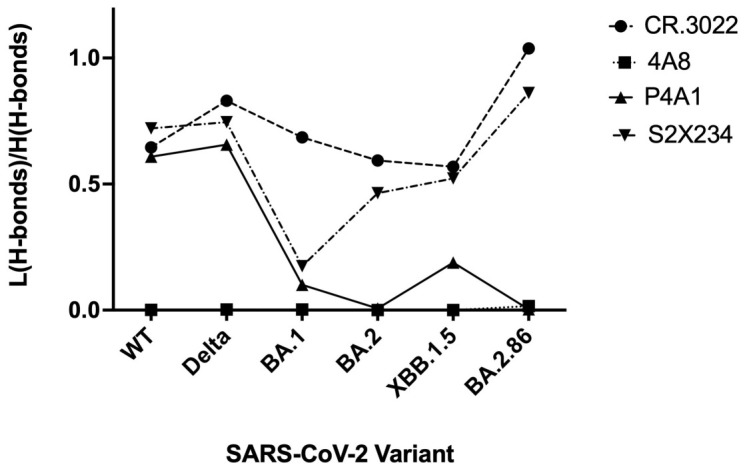
Ratio of interfacial hydrogen bonds to light chain to interfacial hydrogen bonds to heavy chain for selected antibodies. The ratio of the number of interfacial hydrogen bonds from spike domain-to-light chain relative to spike domain-to-heavy chain is plotted for the CR.3022 (Class III), 4A8 (NTD), P4A1 (Class I), and S2X234 (Class I) Abs. For the CR.3022 and S2X234 Abs, viral evolution favors higher relative binding to the light chain, while for the P4A1 Ab, light chain bonding decreases relative to the heavy chain. For the NTD Ab 4A8, light chain bonding is negligible for all studied variants. Graphing was performed using GraphPad Prism version 11.0.0 for MacOS, GraphPad Software, Boston, MA, USA, www.graphpad.com accessed on 15 April 2026.

Population analyses, presented comprehensively in the online data, show that in every case, the majority of the interfacial hydrogen bonds are to the heavy chain of the antibody, although in a few cases, the bond strength to the light chain becomes comparable with viral evolution (for CR 3022, S2X234, C1A-B3, and 2-15 the light chain interfacial hydrogen bond count for the BA.2.86 is comparable to the heavy chain count, while it is significantly smaller for the WT). For the NTD antibody 4A8, there is negligible light chain binding for all variants. We show four plots of the trends of the ratio in Figure 4.

There are no clear systematics about changing of hydrogen bonds with viral evolution for a given antibody. We do find in some cases that interfacial hydrogen bonds are conserved or nearly conserved throughout viral evolution:

- **P4A1** For the P4A1 Ab, hydrogen bonds between the side chain of D420 on the RBD to S56 on the heavy chain are preserved, and between N487 on the RBD and R97 on the heavy chain are preserved. One bond gains considerable strength (population) for omicron and descendants: the S53 of the heavy chain donates to the main chain R457 of the RBD.
- **C1A-B12** Side chain hydrogen bonds between R94 of the heavy chain and N487 of the RBD generally increase with mutation (except for the BA.2 variant). Side chain bonding between Y473 of the RBD and the main chain of S31 in the heavy chain is conserved apart from the XBB.1.5 variant.
- **C1A-C2** Side chain–side chain binding of the T56 heavy chain residue to D420 of the RBD is highly conserved. Side chain–side chain binding of the R94 or R97 heavy chain residues to N487 of the RBD is highly conserved. Side chain–main chain binding of the Y473 residue of the RBD to the N31 of the heavy chain is conserved apart from the BA.1 variant.
- **C1A-F10** Side chain–side chain bonds between R94 or R97 of the heavy chain and N487 of the RBD are strongly conserved. Side chain–side chain bonding between Y473 of the RBD and S31 of the heavy chain are highly conserved. Side chain to side chain or main chain bonding of the R403 residue of the RBD to the N92 residue of the light chain is conserved through XBB.1.5, but K403 of the BA.2.86 variant does not strongly bind to the light chain.
- **C1A-B3** Side chain–side chain bonding of the R97 or R94 of the heavy chain to the N487 of the RBD is conserved apart from BA.1.
- **Omi-3** Heavy chain S56 to RBD N420 side chain–side chain hydrogen bonding is conserved with variants. So is heavy chain R97 to RBD N487 side chain–side chain binding, as well as Y473 of the RBD side chain to main chain R31 of the heavy chain.
- **S2X234** Salt bridge bonding of the K444 RBD residue to the heavy chain D56 and D58 residues is conserved throughout. Side chain binding of the R60 heavy chain residue to the main chain of the G447 RBD is strong throughout the viral evolution. The T500 residue of the RBD exhibits significant main chain bonding to the main chain atoms of N32 of the light chain (apart from BA.1).

In summary, for the strongly binding antibodies P4A1, C1A-B12, C1A-C2, C1A-F10, C1A-B3, and Omi-3, the N487 residue of the RBD plays a key role for binding to the heavy chain. For the P4A1 and Omi-3, the N420 residue of the RBD plays a key role for heavy chain binding. For the C1A-B12, C1A-C2 antibodies, the Y473 residue of the RBD plays a key role for heavy chain binding.

The predominant occupancies for each antibody and each variant are presented in the online data.

### 3.4. Comments on Structural Assumptions

The validity of these results is dependent upon two key experimental assumptions. First, the possibility that N-linked glycans interfere with the antibody binding is not considered, because our simulations were carried out without the attached glycans. Second, we assume a perturbative approach to connect the strains is appropriate, i.e., that by adding mutations to the RBD or NTD from the appropriate starting structure and simulating to equilibirium we can achieve a reasonable representation of the actual bound antibody–viral domain complexes.

Regarding the glycans, we have confidence that, for the Class I and Class III antibodies binding to the RBD, the glycans would have little to no effect upon the simulations. The RBD possesses only two glycans of relevance, as mentioned above, at N331 and N343. Neither is close to the binding interfaces for the antibodies.

The situation for the NTD binding is different. The N149 is directly at the binding interface. Accordingly, the estimates for interfacial hydrogen bond counts and the magnitude of the binding free energy must be considered as upper bounds for the actual values, as the glycan is likely to interfere with the NTD to antibody binding by shielding this region of the spike protein.

Regarding the perturbation analysis, this assumption can be tested experimentally by studying antibody binding structures across the assembled variants. While one might have expected this to be the case, in scanning the protein data bank, we were able to identify only one example of where the original Wuhan strain RBD was bound to the same antibody as a subsequent strain: the 19–77 antibody was bound to the Wuhan RBD and to the omicron descendent HK.3 RBD [27]. To test the perturbative hypothesis, we uploaded both structures to YASARA and performed a sequence alignment using MUSTANG [28] on the two RBD molecules, comparing the C-α alignment of the protein chain. Despite only having an 88% sequence identity, the root mean square deviation (RMSD) between the structures was a remarkably small 0.66 Å. This remarkably close backbone alignment offers partial, if limited support, for the perturbative analysis.

## 4. Discussion

Unsurprisingly, this work shows a general diminution of binding by antibodies developed at a given time to the SARS-CoV-2 spike protein. Surprisingly, for many antibodies we have studied here, there is a modest re-entrant behavior to the binding strength utilizing interfacial hydrogen bond count as a proxy per the correlation between the computed binding energy and the interfacial hydrogen bond count. Speculatively, this can be attributed to an evolutionary drive to achieve antibody escape while maintaining reasonable binding of the receptor binding domain to the ACE2. However, the N-terminal antibody 4A8 and the Class III antibody CR 3022 show modest re-entrance This is subject to the caveat that our studies approached binding perturbatively from the original Wuhan strain of SARS-CoV-2 rather than entertaining a fully new binding motif. In all cases observed in simulation here, the re-entrant behavior is statistically significant as measured by *t*-test *p* values.

This re-entrant immunity result is testable experimentally in affinity studies of antibody binding to SARS-CoV-2 variant spike proteins. When coupled with structural determinations from which interfacial hydrogen bond counts can be made, an experimental test can be made of the correlation between binding free energy (log of the dissociation constant) and interfacial hydrogen bond count.

The significance of the re-entrant immunity is clear. Immunity gained from vaccination or earlier infection is not wholly surrendered, and newer antibodies developed to later variants or later vaccinations can maintain some efficacy against subsequent viral mutants. Meanwhile, since it is virtually impossible to strongly evolve away from Class I antibodies while maintaining sufficient ACE2 receptor binding to enter cells, there is general expectation for the potential damage by the virus to diminish with time, as discussed in previous work [1].

## Figures and Tables

**Table 1:** Mutations in variants relative to original wild-type(WT)strain including the D614G mutation. Column 1 lists the variant, Column 2 mutations in the receptor binding domain (RBD), and Column 3 mutations in the N-terminus. Mutations in the ACE2 binding region of Column 2 are identified in bold print. Point residue mutations were represented by XNY, where X is the WT residue, N the sequence number in the WT, and Y the variant residue. Deletions were represented by Δ(N-M), where *N* is the starting sequence number, *M* is the ending sequence number, and additions were represented by Σ(N−M) similarly. Sequences are listed at Ref. [2], with the original spike sequence at Ref. [3].

Variant	RBD Mutations	N-Terminus Mutations
Delta (B.1.617.2)	**L452R**, **T478K**	T19R, T95I, G142D, Y145H,
		Δ(156-157), F158G, A222V,
		W258L
BA.1	G339D, S371L, S373P, S375F,	A67V, Δ(69-70), T95I, G142D,
	**K417N**, **N440K**, **G446S**, **S477N**,	Δ(143-145), N211K, Δ(212),
	**T478K**, **E484A**, **Q493R**, **G496S**,	Σ(R214)
	**Q498R**, **N501Y**, **Y505H**	
BA.2	G339D, S371F, S373P, S375F,	T19I, L24S, Δ(25-27), G142D,
	T376A, **D405N**, **R408S**, **K417N**,	V213G
	**N440K**, **S477N**, **T478K**, **E484A**,	
	**Q493R**, **G496S**, **Q498R**, **N501Y**,	
	**Y505H**	
XBB.15	G339H, R346T, L368I, S371F,	T19I, L24S, Δ(25-27), G142D,
	S373P, S375F, T376A, **D405N,**	Δ(144), H146Q, Q183E, V213E,
	**R408S**, **K417N**, **N440K**, **V445P**,	G252V
	**E484A**, **G446S**, **N460K**, **S477N**,	
	**T478K**, **F486P**, **F490S**, **Q498R**,	
	**N501Y**, **Y505H**	
BA.2.86	G339H, K356T, S371F, S373P,	T19I, R21T, L24S, Δ(25-27),
	S375F, T376A, **R403K**, **D405N**,	S50L, Δ(69-70), V127F, G142D,
	**R408S**, **K417N**, **N440K**, **V445H**,	Δ(144), F157S, R158G, N211I,
	**G446S**, **N450D**, **L452W**, **N460K**,	Δ(212), V213G, L216F, H245N,
	**S477N**, **T488K**, **N481K**, **Δ(483)**,	A264D, I332V
	**E484K**, **F486P**, **Q498R**, **N501Y**,	
	**Y505H**	

**Table 2:** Antibodies by class studied here, with class in column 1, antibody nomenclature in column 2, relevant PDB entry in column 3.

Antibody Class	Antibody Label	PDB Entry
Class I	P4A1	7CJF
Class I	C1A-B12	7KFV
Class I	C1A-C2	7KFX
Class I	C1A-F10	7KFY
Class I	C1A-B3	7KFW
Class I	2-15	7L5B
Class I	S2X234	8ERQ
Class I	Omi-3	7ZF3
Class III	CR.3022	6YOR
N-term	4A8	7C2L

## Data Availability

Data for simulations, analyses, and hydrogen bond populations is available at https://drive.google.com/drive/folders/1ZZg0VOnmag9k8El5af459iiH_AzPapdz?usp=sharing. This folder was accessed on 17 April 2026.
